# Protein–lipid co‐oxidation in emulsions stabilized by microwave‐treated and conventional thermal‐treated faba bean proteins

**DOI:** 10.1002/fsn3.641

**Published:** 2018-04-16

**Authors:** Göker Gürbüz, Chang Liu, Zhong‐qing Jiang, Marjo Pulkkinen, Vieno Piironen, Tuula Sontag‐Strohm, Marina Heinonen

**Affiliations:** ^1^ Department of Food and Nutrition University of Helsinki Helsinki Finland

**Keywords:** emulsions, faba beans, lipid oxidation, lipoxygenase, protein oxidation

## Abstract

The course of protein–lipid co‐oxidation was investigated in oil‐in‐water emulsions stabilized with proteins extracted from microwave‐treated (MWT) and conventional thermal‐treated (CTT) faba beans stored at 37°C for 7 days. Emulsions prepared with proteins from untreated (UT) faba beans and soy protein isolate (SP) were monitored for comparison. Lipid oxidation was detected through formation of primary and secondary oxidation products while protein oxidation was examined via tryptophan fluorescence degradation in interface and aqueous phase. Oxidation of proteins was more emphasized in the interfacial layers of MWT, CTT, and SP emulsions than in UT emulsions due to the prominence of radical chain‐driven co‐oxidation mechanism while lipoxygenase (LOX) activity in UT and MWT emulsions resulted in high amounts of hydroperoxides and abundance in lipid oxidation volatiles. Conventional thermal treatment provided better oxidative stability than microwave treatment reflected in lower levels of hydroperoxides and relative lack of diversity in lipid volatiles. Among detected volatiles, formation of ketones was more distinguished in MWT, CTT, and SP emulsions while UT emulsions contained a more diverse range of alkenals and alkanals. Ketones are known to form mainly through radical recombination reactions which combined with the results of protein oxidation supports that radical transfer reactions between proteins and lipids were the driving force behind oxidation in MWT, CTT, and SP emulsions. Treatments of faba beans resulted in increased oxidative stability of emulsified lipids and lower degradation of aqueous phase proteins.

## 1 INTRODUCTION

Oxidative stability of food emulsions is one of the major concerns in terms of shelf‐life reduction due to loss of nutritional quality, undesired structural alterations, and sensory deterioration. While lipid oxidation is an extensively researched topic, protein oxidation and its interactions with lipids and their oxidation products remain relatively understudied. In oil‐in‐water type of emulsions, oil droplets are covered by an interfacial membrane of surface‐active molecules preventing the droplets aggregate and thus, characteristics of the interfacial layer play a key role in overall properties of the emulsion including the course of lipid oxidation (Frankel, 2005; McClements, 2004). Proteins are commonly used food emulsifiers owing to their surface‐active amphiphilic nature. Expectedly, their characteristics such as isoelectric point, conformational structure, amount of free sulfhydryl groups, play an important part on oxidative stability. Protein oxidation and lipid oxidation have been reported to advance in an interdependent relationship where proteins react with free radicals (Schaich, 2008), lipid hydroperoxides (Wu, Hou, Zhang, Kong, & Hua, 2009), and oxidation‐derived aldehydes (Doorn & Petersen, 2002; Gürbüz & Heinonen, 2015) while they also can act as pro‐ or anti‐oxidants (Ries, Ye, Haisman, & Singh, 2010). Several studies have found that the effect of proteins on lipid oxidation is not only limited to their adsorbed location on the interface but also to their presence in the continuous phase where unadsorbed proteins have been reported to contribute to antioxidant activity against lipid oxidation via metal‐chelating and free radical scavenging mechanisms (Cheng, Xiong, & Chen, 2010; Elias, McClements, & Decker, 2007; Faraji, McClements, & Decker, 2004).

Faba bean (*Vicia faba* L.)—also known as broad bean, fava bean, horse bean and field bean—is a rich source of protein which can be cropped in diverse climates ranging from temperate and subtropical to boreal regions (Multari, Stewart, & Russell, 2015). However, proteins of faba beans and other legumes—with the exception of soybean—remain nutritionally and industrially underexploited. Major faba bean proteins include globulin, albumin, prolamin, and glutelin fractions with globulin comprising more than 60% of total proteins (El Fiel, El Tinay, & Elsheikh, 2002). Functional properties of faba bean proteins in emulsions have been studied via various methods of preparation. Sosulski and McCurdy (1987) reported high emulsifying and solubility levels of faba bean protein fractions while Krause, Mothes, and Schwenke (1996) studied the relationship between acetylation of faba bean legumin and interfacial activity in emulsions, concluding that acetylation increased the emulsifying properties. Makri, Papalamprou, and Doxastakis (2005) used faba bean proteins prepared by isoelectric precipitation and ultrafiltration in order to characterize emulsion stability in the presence of xanthan gum. Moreover, Karaca, Low, and Nickerson (2011) included faba bean proteins in a study to differentiate the emulsifying properties of isoelectrically precipitated and salt‐extracted proteins. Apart from their functional use, utilization of faba bean proteins also depends on their effect on sensory attributes upon which lipoxygenase activity would have a profound impact due to its ability to trigger lipid oxidation rapidly. Lipoxygenase enzyme, which is naturally present in plants, targets polyunsaturated fatty acids. Enzyme‐catalyzed lipid oxidation is responsible for prompt formation of hydroperoxides which decompose into numerous volatiles flavor compounds (Jeleń & Wąsowicz, 2012; Ties & Barringer, 2012).

In this study, the effects of microwave and conventional thermal treatment on the faba bean proteins in terms of oxidative stability of protein‐stabilized oil‐in‐water emulsions were investigated. Our aim was to study the course of oxidation in a multiphase food model system in which faba bean proteins were utilized as emulsifier in order to investigate the significance of co‐oxidation of protein and lipid components.

## 2 MATERIALS AND METHODS

### 2.1 Materials

Beans used in the study belonged to “Kontu” cultivar grown at Viikki experimental farm (Lizarazo et al., 2014). Conventional thermal and microwave treatments on the beans were carried out as reported by Jiang et al. (2016). Conventional thermal treatment (CTT) referred to heating faba beans with an air oven at 170°C for 30 min. In order to avoid excessive water evaporation, the beans were packed in a sealed glass bottle during heating in triplicates. The microwave treatment (MWT) was conducted by heating 200 g of faba beans with a microwave oven (microwave frequency 2450 MHz, Whirlpool JT‐379, USA) at 950 watts for 1.5 min in five replicates. The treatments caused a moisture content decrease from 11.8% to around 10% in MWT and around 10.5% in CTT samples. After the treatments, beans were dehulled with a stone mill and ground with a high speed rotor ultracentrifugal mill (Ultra Centrifugal Mill ZM 200, Retsch, Germany, sieve pore size 0.5 mm) into fine flour.

Rapeseed oil was acquired from a local store (Keiju Rypsiöljy, Bunge Finland Oy, Raisio, Finland). Soy Protein Isolate (Supro^®^ EX 45 IP) was purchased from SolaeTM, LLC (St. Luois, MO, USA). Albumin from bovine serum, linoleic acid (≥99%), sodium dodecyl sulfate (≥99%, GC), Tween^®^20, 2‐propanol (CHROMASOLV^®^, for HPLC, 99.9%), 1,4‐dioxane (≥99.5%), and heptane (CHROMASOLV^®^, for HPLC, ≥99%) were purchased from Sigma‐Aldrich Chemie GmbH (Steinheim, Germany) while soy milk with vanilla flavor (Alpro C.V.A., Wevelgem, Belgium) to be used as an in‐house reference for SPME‐GC‐MS method was purchased from a local store. Tocopherol standards (α‐, β‐, γ‐, δ‐), aluminum oxide (Al_2_O_3_, 90 active neutral, activity stage I, for column chromatography, 0.063–0.200 mm, 70–230 mesh ASTM), sodium hydroxide pellets (NaOH), disodium hydrogen phosphate dihydrate (Na_2_HPO_4_ ∙ 2H_2_O), sodium dihydrogen phosphate monohydrate (NaH_2_PO_4_ ∙ H_2_O) and sodium azide (NaN_3_) were acquired from Merck (Darmstadt, Germany). Water used throughout the study was purified via Milli‐Q equipment (Millipore Corp., Bedford, MA, USA).

### 2.2 Lipoxygenase activity measurement

Lipoxygenase (LOX) activity measurement in faba bean flours was based on methods described by Axelrod, Cheesbrough, and Laakso (1981), Gökmen, Bahçeci, and Acar (2002) and Jiang et al. (2016). Enzyme was extracted by mixing 20 ml of water with 0.5 g of flour and shaking thoroughly for 15 min followed by centrifugation for 10 min at 10,000 rpm. Then, aqueous part was collected and filtered through a filter paper to be used as the enzyme extract. Equal amounts of linoleic acid and Tween^®^20 were mixed with water to form a stable emulsion, and 300 μl of 1 N NaOH was added to clarify the solution. Afterward, 0.2 ml of linoleate substrate (10 mmol/L) was added to 2.6 ml 0.1 mol/L sodium phosphate buffer (pH 6.0) and mixture was placed in a water bath of 25°C. Subsequently, 0.2 ml of enzyme extract was added into the mixture to start the enzymatic reaction and kept on proceeding for 5 min before 3 ml of 0.1 N NaOH solution was added to the mixture to conclude the reaction. For blank samples, the same procedure was followed in which 0.2 ml water was added to substrate mixture instead of the enzyme extract. Afterward, ultraviolet absorbance of conjugated dienes (CD) in samples was measured at 234 nm using a UV/Vis spectrophotometer (Lambda 25; PerkinElmer Inc., MA, USA). LOX activity in samples was expressed as μmol CD per g sample per minute with molar absorptivity value of 25,000 cm^−1^ mol/L^−1^.

### 2.3 Extraction of water‐soluble proteins

Flour samples of untreated, microwave‐treated and conventional thermal‐treated faba beans were mixed with water with a ratio of 1:7 (w/v) and left to shake for one hour at room temperature in an oscillator (Grant OLS200, Grant Instruments, UK) at a speed of ~150 strokes/min. Mixture was then centrifuged for 20 min at 10,000 rpm at ~10°C. Afterward, the aqueous part was collected and passed through a filter paper. Protein content of the water‐soluble protein extracts from different flour samples was then determined using a protein assay kit (Bio‐Rad DC^™^ Protein Assay, CA, USA). Bovine serum albumin was used to obtain a standard curve.

### 2.4 Preparation of emulsions

Rapeseed oil to be used in emulsions was stripped of its tocopherols according to the method described by Lampi, Dimberg, and Kamal‐Eldin (1999) with modifications. A glass column (51 cm × 2.9 cm i.d.) was packed with 180 g activated aluminum oxide (kept at 100°C for 16 hr, then at 200°C for 8 hr) and conditioned with heptane. Later, 100 g oil dissolved in 100 ml heptane was eluted in order to dispose of tocopherols, pro‐oxidants, and trace metals. Purified oil was then stored in heptane at −20°C until further use in emulsion preparation. Normal‐phase HPLC connected with a fluorescence detector was used to check the residual tocopherols according to the method described by Schwartz, Ollilainen, Piironen, and Lampi (2008). The results showed no detectable residues of tocopherols.

Oil‐in‐water emulsions used in the study were prepared with adjusted final concentrations of 3% (w/v) faba bean or soy proteins and 10% (w/v) purified rapeseed oil. Before use, heptane portion of the oil‐in‐heptane solution was evaporated under nitrogen flow followed by addition of protein extracts in water and a brief pre‐homogenization procedure using an Ultra‐Turrax^®^ T25 homogenizer (IKA^®^‐Werke GmbH & Co. KG, Germany). Next, stable oil‐in‐water emulsions were achieved using an M‐110Y Microfluidizer^®^ processor (Microfluidics^™^, MFIC Corp., MA, USA) at an operating pressure of 600 bars for 10 min of continuous flow process. Each emulsion sample was divided into three replicates from which analytical samples were taken on days 0, 1, 4, and 7 of the oxidative storage period. Finally, sodium azide was added with a final concentration of 0.02% (w/v) to prevent possible microbial growth. Sample vials each containing around 13 ml of emulsion and closed with caps were then placed at 37°C in dark with magnetic stirring.

### 2.5 Monitoring oxidation

In order to monitor oxidative changes, aliquots were taken from emulsion samples on days 0, 1, 4, and 7. Lipid oxidation was surveilled through spectrophotometric measurement of conjugated diene hydroperoxides (CD) as primary oxidation products and detection of secondary lipid oxidation volatiles via headspace solid‐phase microextraction–gas chromatography–mass spectrometry (HS‐SPME‐GC‐MS). Conjugated diene measurements were performed according to method described by Lethuaut, Métro, and Genot (2002) with slight adjustments. Aliquots from emulsions were mixed with 5 ml of 2‐propanol which was followed by centrifugation at 6500 rpm for 15 min. Absorbance of supernatant portion was spectrophotometrically measured at 234 nm and results were expressed as mmol CD/kg oil.

Secondary lipid oxidation products were observed as integrated peak areas of selected volatile compounds which were used to compare the progress of oxidation in an emulsion between sampling days rather than quantification of these volatiles. Emulsion aliquots of 1.5 ml were collected on sampling days in HS‐SPME‐GC vials. The equipment set‐up comprised an SPME injector (combiPAL; CTC Analytics, USA), a GC (HP 6890 series; Agilent Technologies Inc., DE, USA), and a MS detector (Agilent 5973 Network; Agilent Technologies Inc.). The volatiles were extracted using a divinylbenzene/ carboxen/ polydimethylsiloxane (DVB/CAR/PDMS) SPME fiber assembly with 50/ 30 μm film thickness (Stableflex 23 Ga, Supelco, PA, USA). Chromatographic separation of compounds was achieved through a SPB^®^‐624 capillary column with dimensions of 30 m × 0.25 mm i.d. and 1.4 μm film thickness (Supelco, PA, USA). The SPME‐GC‐MS method employed in the study was developed by Damerau, Kamlang‐ek, Moisio, Lampi, and Piironen (2014). Equilibration step was carried out at 40°C for 10 min with an agitator speed of 250 rpm followed by extraction at 40°C for 30 min. Next, fiber was desorbed for 10 min at 250°C at the GC front inlet in “spitless” mode. GC was operated with a helium flow of 0.7 ml/min while temperature gradient of the GC oven was set as follows: 40°C for the first two min, then incremental increase at a rate of 5°C/min until 200°C followed by an 11 min of fixed temperature of 200°C. Ionization energy for MS detection was 70 eV and m/z scan range was 40–300 amu. Identification of the volatile compounds was based on mass spectral data library Wiley 7N (Wiley Registry^™^ of Mass Spectral Data, 7th ed., USA) and retention times of these compounds in previously published data (Damerau et al., 2014).

Modifications to proteins were examined via changes in tryptophan fluorescence of proteins that were located both in continuous phase (unadsorbed proteins) and lipid interface (adsorbed proteins). Sample aliquots collected in Eppendorf^®^ tubes were put to centrifugation at 14,000 rpm for 20 min to separate phases in the emulsion. Subsequently, part of the aqueous fraction was taken for fluorescence measurements while an aliquot of the cream layer was collected to a different container. In order to separate the adsorbed proteins from the lipid interface, 1% SDS solution was added to the collected cream portion (Chapleau & de Lamballerie‐Anton, 2003) and centrifuged at 9500 rpm for 15 min. As a result of the newly separated phases, aqueous portion contained the adsorbed proteins of initial emulsion. An aliquot of this phase was collected for fluorescence spectroscopy. Protein content measurement of unadsorbed proteins and adsorbed proteins revealed that approximately 10–15% of the faba bean proteins were situated at the interfacial layer. Tryptophan fluorescence in both phases was monitored using a fluorometer (LS 55 Luminescence Spectrometer, PerkinElmer Inc., MA, USA) to scan the emissions between 300 and 400 nm upon excitation at 283 (Estévez, Kylli, Puolanne, Kivikari, & Heinonen, 2008). Changes in tryptophan fluorescence were calculated in terms of percentage with respect to initial fluorescence intensity on day 0.

## 3 RESULTS AND DISCUSSION

### 3.1 Lipoxygenase activity in faba bean flours

Both microwave (MWT) and conventional thermal treatments (CTT) had a remarkable effect on reducing the lipoxygenase activity in faba bean flours compared to untreated faba bean samples (UT). CTT caused a 96% inhibition of lipoxygenase (LOX) activity, while MWT ended up decreasing the activity around 77% (Table 1). Jiang et al. (2016) reported that both treatments were also effective in inhibition of the native peroxidase enzyme present in faba bean samples.

**Table 1:** Lipoxygenase activity measured in faba bean flours

Sample name	Lipoxygenase activity (±SD^a^) (μmol CD^b^ / g sample/ min)	% inhibition of native enzyme
Untreated faba bean flour	56.5 ± 0.9	
Microwave‐treated faba bean flour	12.9 ± 1.9	77.3
Heat‐treated faba bean flour	2.3 ± 0.1	96.0
Control sample	1.4 ± 0.6	

a SD: Standard deviation.

b CD: Conjugated diene hydroperoxides.

LOX acts on *cis*, *cis*‐1,4‐pentadiene structures, namely linoleic and linolenic acids, as substrates and not on oleic acid. Faba bean lipoxygenase catalyzes the direct formation of hydroperoxides 9‐hydroperoxyoctadecadienoic (9‐OOH) and 13‐hydroperoxyoctadecadienoic (13‐OOH) acids from linoleic acid without generating radicals (Schaich, 2005). Furthermore, the activation energy necessary to oxidize linoleic acid is much lower in LOX‐catalyzed reactions compared to the photooxidation/autoxidation pathway (Belitz, Grosch, & Schieberle, 2009) which makes the enzymatic pathway favorable over autoxidation. Even though the optimum conditions for the highest activity of faba bean lipoxygenase were found to be 30°C and pH 6.0, Al‐Obaidy and Siddiqi (1981) reported that the enzyme did not lose its activity up to 55°C and had a wide range of stability between pH 4.0 and pH 8.0. Thus, LOX activity was anticipated to have a dominant effect on formation of oxidation products in untreated faba bean protein emulsions compared to other emulsions.

### 3.2 Lipid oxidation products

**Figure 1:**
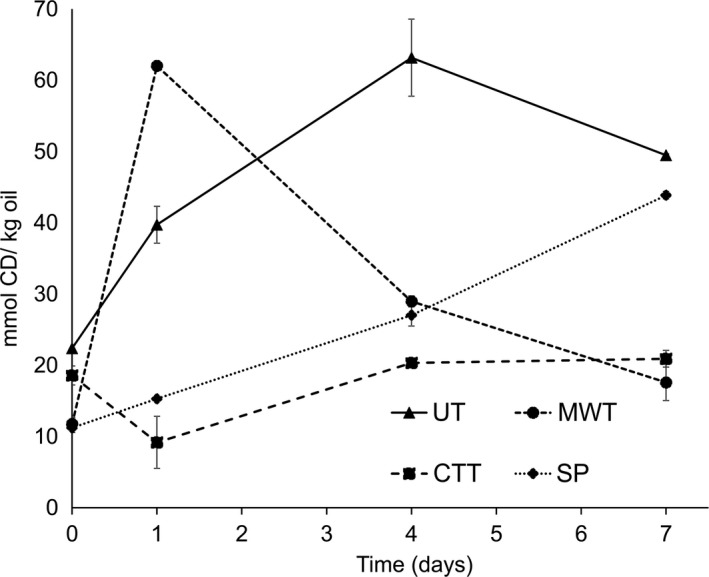
Formation of conjugated dienes (CD) during 7 days of storage in emulsions stabilized with proteins from untreated (UT), microwave‐treated (MWT), conventional thermal‐treated (CTT) faba beans and soy protein isolate (SP)

**Figure 2:**
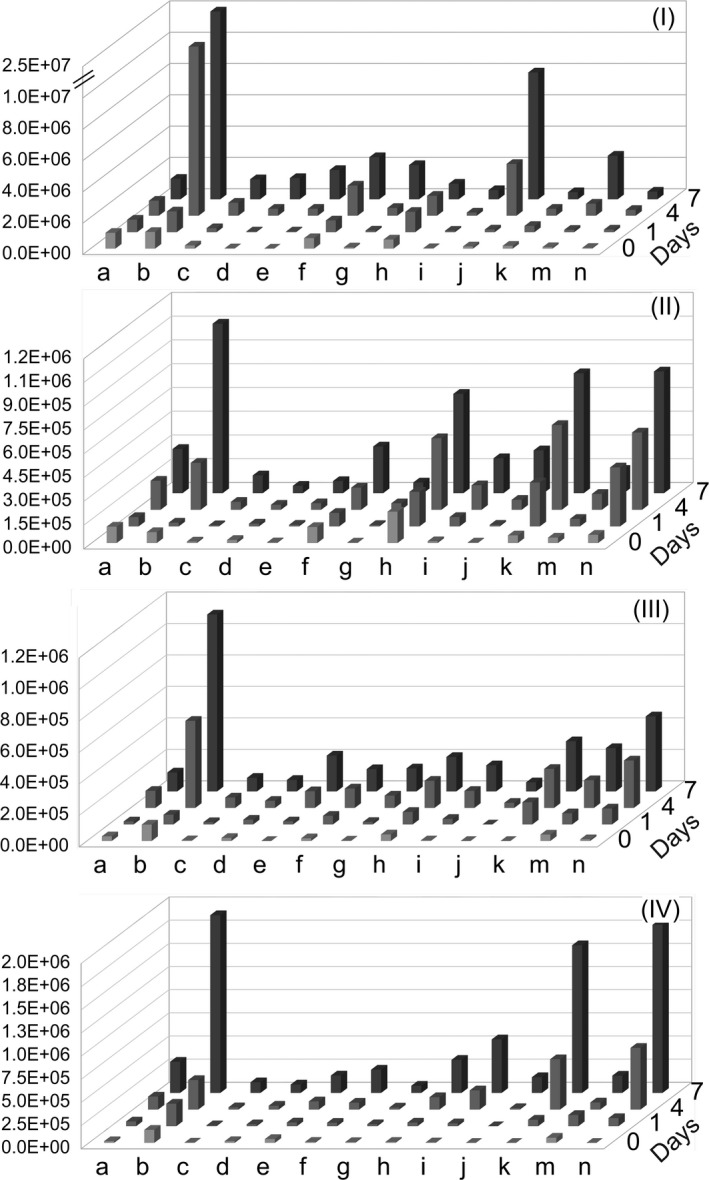
Formation of lipid oxidation volatiles observed in chromatographic peak areas during 7 days of storage as detected by SPME‐GC‐MS in (I) untreated, (II) microwave‐treated, (III) conventional thermal‐treated faba bean and (IV) soy protein emulsions. Volatiles denoted on x‐axis by lower case letters as, a: (E)‐2‐pentenal, b: hexanal, c: (E)‐2‐hexenal, d: (E)‐2‐heptanone, e: 2‐pentylfuran, f: (E)‐2‐heptenal, g: octanal, h: (E,E)‐2,4‐heptadienal, i: (E)‐3‐octen‐2‐one, j: (E)‐2‐octenal, k: (E,Z)‐3,5‐octadien‐2‐one, m: nonanal, n: (E,E)‐3,5‐octadien‐2‐one

Conventional thermal treatment of faba beans resulted in improved oxidative stability of the emulsified lipids with respect to formation of lipid oxidation products (Figure 1, Figure 2). On the contrary, the highest amounts of hydroperoxides were detected in MWT and UT emulsions within one day of storage at 37°C (Figure 1). Rapid CD formation observed in MWT emulsions turned into a high‐rate decomposition trend after day 1. The initial surge of CD generation could be the combined result of remaining LOX activity and the free radical transfer reactions from the reactive protein species that are formed during microwave treatment. In UT emulsions, the dominant enzymatic oxidation pathway produced a high yield of hydroperoxides already during sample preparation, however, after day 4, the remarkable decomposition of accumulated hydroperoxides into volatiles leads to lower levels of CD concentration as measured at day 7. On the other hand, emulsions prepared with soy protein isolate (SP) were found to exhibit an increasing concentration of hydroperoxides during oxidative storage, the highest rate of formation being observed in the last days. This behavior was also reflected in the volatile detection in these emulsions which indicates a more continuous propagation of lipid oxidation mechanism where observation of stable end products was relatively delayed compared to faba bean protein‐stabilized emulsions.

Among the volatile compounds detected via SPME‐GC‐MS, prominent ones were selected to be monitored as oxidation products. These compounds consisted of aldehydes (E)‐2‐pentenal, hexanal, (E)‐2‐hexenal, (E)‐2‐heptenal, octanal, (E,E)‐2,4‐heptadienal, (E)‐2‐octenal, nonanal, ketones (E,Z)‐3,5‐octedien‐2‐one, (E)‐2‐heptanone, (E,E)‐3,5‐octadien‐2‐one, (E)‐3‐octen‐2‐one, and alkylfuran 2‐pentylfuran. Treatments of faba beans, in particular conventional thermal treatment, resulted in improved oxidative stability toward formation of volatile lipid oxidation products (Figure 2). Advanced stages of oxidation were observed in UT emulsions as expected due to high LOX activity. In particular, volatiles marking advanced oxidation such as 1‐pentanol, 1‐octen‐3‐ol, 1‐heptanol, hexanoic acid, octanoic acid (Lampi et al., 2015) and longer chain aldehydes (E,E)‐2,4‐nonadienal and (E,E)‐2,4‐decadienal were found only in UT emulsions. As the most common oxidation product of linoleic acid and an established marker of lipid oxidation, hexanal was the most prominent compound observed in all samples. On the other hand, the share of detected volatiles showed variation in abundance within different emulsions groups. For instance, UT emulsions were dominated by linoleic acid‐originating (E)‐2‐octenal and (E)‐2‐heptenal (Schaich, 2005) while ketones were more prevailing in MWT, CTT, and SP emulsions. This could be explained by the formation pathway of ketones which is through radical recombination reactions that involve alkyl, peroxyl, and alkoxyl radicals, while LOX catalyzes hydroperoxide formation without generating radicals (Schaich, Shahidi, Zhong, & Eskin, 2013). In addition to ketones, another prevailing volatile observed among others in MWT, CTT, and SP emulsions was (E,E)‐2,4‐heptadienal which is known to be a major decomposition product of 12‐linolenate hydroperoxide (Frankel, 1982). Although it was also detected in highly oxidized UT emulsions, it was not as favored as a dominant product as in the other emulsions. Diversity of volatiles found in higher abundances in MWT emulsions was broader than in CTT emulsions. These compounds included 2‐pentenal and 2,4‐heptadienal which are oxidation products of linolenic acid and linolenic acid‐derived volatiles 2‐octenal, 2‐heptenal, and 3‐octen‐2‐one (Damerau et al., 2014; Frankel, 1982). Generation of 2‐pentylfuran, another linoleic acid volatile, was more emphasized in CTT emulsions than in MWT emulsions, while major linoleic acid product hexanal was detected in comparable levels in both emulsions. Even though 2‐pentylfuran formation was more evident in CTT emulsions, generation of wider range of linoleic acid products detected in MWT emulsions indicates that this fatty acid was utilized as a substrate both by LOX activity and radical reactions resulting in higher diversity of linoleate volatiles. Accordingly, in MWT emulsions, this broader range of volatiles was a reflection of ample hydroperoxide formation in comparison with CTT emulsions. Thus, conventional thermal treatment was a more efficient means of containing the progress of oxidation than microwave treatment.

### 3.3 Protein oxidation

**Figure 3:**
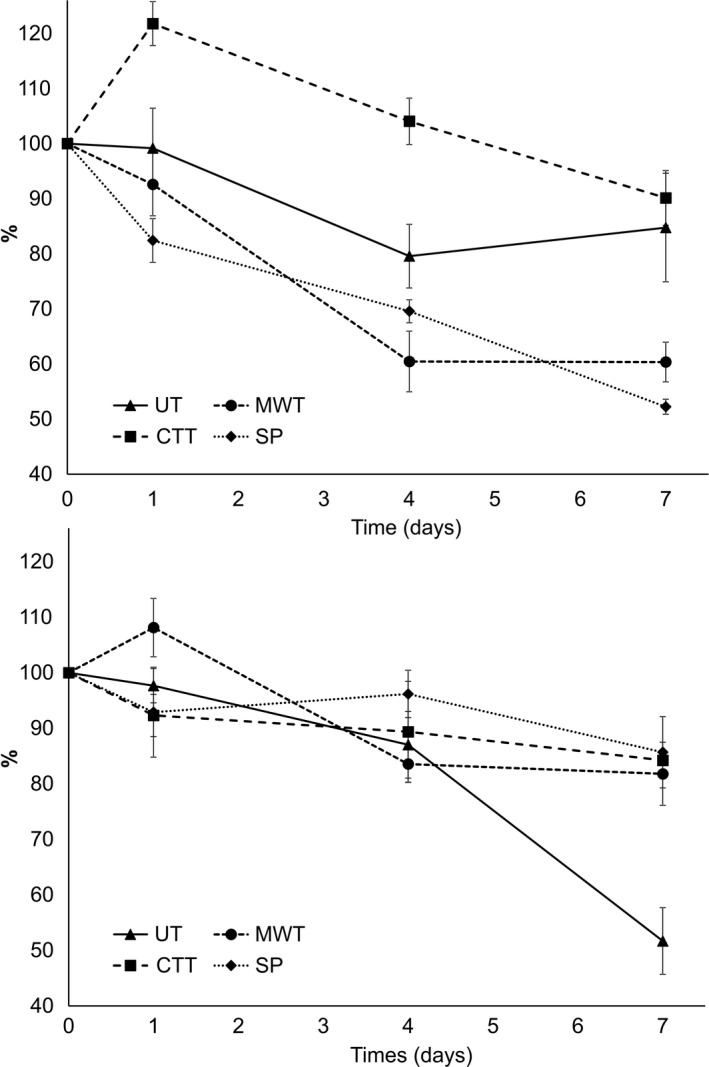
Changes in tryptophan fluorescence in adsorbed (upper) and unadsorbed (lower) proteins presented as percentage of initial day fluorescence intensity from emulsions stabilized with untreated (UT), microwave‐treated (MWT), conventional thermal‐treated (CTT) faba bean proteins and soy protein isolate (SP)

Tryptophan fluorescence was monitored in both phases of the emulsions. Fluorescence peak maxima were found at emission wavelengths around 345 nm in adsorbed proteins and around 332 nm in unadsorbed proteins. The difference in peak maxima wavelengths was also reported previously (Berton, Ropers, Guibert, Solé, & Genot, 2012; Salminen, Heinonen, & Decker, 2010) and is due to the conformational differences in the proteins from aqueous and cream phase and the location of tryptophan in these structures (Munishkina & Fink, 2007). Oxidative degradation of interfacial proteins in MWT emulsions was more advanced compared to CTT emulsions (40% decrease in Trp fluorescence vs. 30% from day 1 in CTT) most likely due to more advanced propagation of lipid oxidation in MWT emulsions (Figure 3). Due to the higher temperature that faba beans were exposed in CTT samples, the degree of denaturation and thus the functionality of proteins stabilizing lipid droplets were expected to differ from those of MWT samples. Fan et al. (2016) have reported that microwave treatment prompted formation of protein radicals in a larger extent than conventional oven heating due to excitation of water molecules that in turn attack proteins. Therefore, it is possible that protein radicals already present in MWT emulsions played a significant role in propagation of both protein oxidation and lipid oxidation. Secondary lipid oxidation products were detected in a higher initial rate in MWT emulsions than CTT and SP emulsions which shows that radical‐driven protein oxidation reactions in MWT emulsions augmented lipid oxidation. Within the first day of emulsion preparation, CTT proteins displayed an increase in tryptophan fluorescence. The reason behind this rise most likely originated from the conformational changes taking place during the thermal treatment. Adsorbed proteins of CTT emulsions may have been in the course of reaching an equilibrium between folded and unfolded states (Eftink, 1994) during the first day of storage while stabilizing the lipid droplets which lead to a delayed intensity of tryptophan fluorescence.

As a general trend, proteins in the lipid–water interface were oxidized more extensively than those in the aqueous phase with the exception of UT emulsions. This outcome was supported by several studies (Berton et al., 2012; McClements & Decker, 2000), suggesting that abundance of reactive lipid species and hydroperoxides in closer proximity to the interface propagates protein oxidation. In UT emulsions, oxidation of unadsorbed proteins was more pronounced than those at the interface. It is possible that the dominant enzymatic oxidation pathway in these emulsions did not rely on the radical chain‐driven autoxidation due to direct hydroperoxide formation which may have hindered the generation of protein radical species and oxidative damage of proteins. In the aqueous phase, unadsorbed proteins of UT emulsions were substantially oxidized only in the advanced stages of the storage experiment. This may be due to the extensive accumulation of LOX‐catalyzed lipid hydroperoxides and secondary oxidation products in the later stages of the storage which are more polar in nature than their lipid source (Schaich et al., 2013) and thus may have migrated to the aqueous phase triggering extensive protein oxidation here. In comparison, SP samples stabilized with a commercial soy protein isolate showed no complexity in terms of progress of oxidation in the emulsions. Both net formation of CD and secondary oxidation products (in particular hexanal and ketones) displayed a steady increase as well as the degree of protein oxidation at the interfacial layer.

## 4 CONCLUSION

Microwave and conventional thermal treatments considerably increased the oxidative stability of emulsions prepared with faba bean proteins. The extent of lipid and protein oxidation was more pronounced in MWT emulsions compared to CTT emulsions. Dominant lipoxygenase (LOX) pathway in UT emulsions resulted in diversity of secondary oxidation volatiles compared to other emulsions. The prominent occurrence of ketones and the pronounced protein oxidation in MWT, CTT, and SP emulsions supported the fact that reactions of radical species were the main oxidative pathway in these emulsions. Moreover, in MWT, CTT, and SP emulsions, protein oxidation was more advanced in the interface than in aqueous phase, while unadsorbed proteins in UT emulsions underwent extensive oxidation during the last days of the storage. Thus, the treatments of faba beans yielded better oxidative stability of lipids and protection against widespread degradation of proteins.

## DISCLOSURE

Authors declare no conflict of interest.
